# Functional profiling of synthetic camel milk-derived peptides with implication in glucose transport and diabetes

**DOI:** 10.1371/journal.pone.0320812

**Published:** 2025-03-28

**Authors:** Irfa Anwar, Farheen Badrealam Khan, Bincy Baby, Priya Antony, Priti Mudgil, Chee-Yuen Gan, Sajid Maqsood, Ranjit Vijayan, Khalid Muhammad, Mohammed Akli Ayoub

**Affiliations:** 1 Department of Biology, College of Science, United Arab Emirates University, Al Ain, The United Arab Emirates; 2 Department of Biological Sciences, College of Medicine and Health Sciences, Khalifa University, Abu Dhabi, The United Arab Emirates; 3 Department of Food Science, College of Agriculture and Veterinary Medicine, United Arab Emirates University, Al Ain, The United Arab Emirates; 4 Analytical Biochemistry Research Centre, University Innovation Incubator Building, SAINS@USM Campus, Universiti Sains Malaysia, Bayan Lepas, Penang, Malaysia; Government College University Faisalabad, PAKISTAN

## Abstract

We previously identified protein hydrolysates from camel milk (CM) targeting dipeptidyl peptidase IV (DPP-IV) and insulin receptor (IR) activity. In this study, we synthesized nine peptides (P1 to P9) derived from such CM hydrolysates and profiled their potential bioactivity *in vitro* and *in silico*. This aims to validate and determine if such synthetic and pure CM-derived peptides are bioactive on IR function and glucose uptake using pharmacological and functional approaches in human embryonic kidney (HEK293) and human hepato-carcinoma (HepG2) cells. Our bioluminescence resonance energy transfer (BRET) results showed a partial activity of most peptides on IR activity in HEK293 cells ranging from 13 ± 1% to 65 ± 4%, and their potency varies from 3.13 ± 1.72 μg/ml to 12.30 ± 5.66 μg/ml. Combining the saturating dose (0.1 mg/ml) of peptides with insulin (1 μM) revealed three different profiles: non-efficient, potentiating, and antagonistic peptides. The potentiating effect of the peptides was from 104 ± 18% to 147 ± 11%, with one peptide (P2) reducing insulin’s response to 52 ± 8%. Moreover, the peptides slightly promoted IR and AKT phosphorylation and glucose uptake in HepG2 cells with an efficacy of 56 ± 9% to 150 ± 18% on glucose transport. Our molecular docking study on the insulin-bound IR complex identified a potential allosteric binding site for specific bioactive peptides. Overall, our data confirmed the bioactivity of the synthetic CM-derived peptides on IR, AKT, and glucose uptake, consistent with the previous study on CM hydrolysates. The synthesis of the peptides and their validation provide further molecular insights into the antidiabetic action of CM. The study should pave the way for further *in vitro* and *in vivo* characterization of the peptides and developing potent and safe antidiabetic drugs based on the different CM-derived peptides described here.

## Introduction

The antidiabetic properties of CM have been consolidated by several *in vitro* and *in vivo* studies [1–4] as well as clinical trials [5–7]. The major gap in the field was the lack of knowledge of the cellular and molecular mechanisms involved and the unknown CM molecule or agent mediating such CM-mediated effects. Indeed, most studies are *in vivo* using animal diabetic models fed with raw CM and analyzing clinical and histological parameters related to diabetic profile. Although they consolidated the benefits of CM in diabetes, these studies did not provide a rational explanation for the exact mode of action of CM and its plausible antidiabetic agent. However, the recent progress in this field, including those brought by our previous studies, led to a better understanding of the molecular and cellular mechanisms of such antidiabetic action of CM. Overall, CM positively acts directly or indirectly on the key pathways involved in the secretion of insulin and incretins, such as glucagon-like peptide 1 (GLP-1), and the well-functioning of the pancreatic β-cells. This was mainly shown to involve anti-inflammatory, anti-oxidative stress, and anti-apoptotic effects of CM in the pancreatic β-cells. Moreover, CM mediates pathways leading to increased insulin sensitivity and action controlling glucose transport and metabolism [8–11].

Our contribution to this research was substantial by dissecting one of the molecular aspects of the regulation of insulin secretion and glucose metabolism by insulin and its receptor (IR) [8–10]. Our previous studies investigated the functional and pharmacological effects of CM-derived proteins/peptides directly or indirectly on dipeptidyl peptidase IV (DPP-IV) and IR activity and signaling at the cellular level [2,8,9,12]. Indeed, we previously demonstrated that CM whey contains proteins that positively modulate IR and its downstream signaling, such as the phosphorylation of extracellular signal-related kinases (ERK1/2) and AKT kinase in HEK293 and HepG2 cells. Moreover, our experimental approach based on CM whey protein fractionation and hydrolysis by digestive enzymes, including trypsin and pepsin, led to identifying heterogenous hydrolysate fractions that contain a mixture of peptides as potential bioactive CM agents targeting DPP-IV, IR, and glucose transport process inside the cells [8,9,11–13]. Indeed, we identified multiple peptides in those trypsin-/pepsin-generated hydrolysates that may be responsible for the dual effect characterized by the inhibition of DPP-IV *in vitro* and the positive modulation of IR activity, downstream kinase phosphorylation, and glucose uptake in HEK293 and HepG2 cells [9,12,14]. This is consistent with the findings that CM is a source of bioactive peptides with beneficial nutritional and medicinal effects [1,4,15]. In addition, CM lactoferrin was shown as one of the putative protein candidates because it reproduced all the effects of raw CM whey proteins and their hydrolysates [10]. Interestingly, CM whey proteins and their hydrolysates showed synergistic/positive allosteric responses when combined with insulin *in vitro* suggesting that the bioactive CM fractions increased cells and receptor sensitivity towards insulin [9]. This can be considered consistent with the adjuvant effect of CM demonstrated in many *in vivo* experiments reporting that feeding type 1-diabetic animals with CM reduced the insulin doses required for injection [1–4]. Our approach based on CM hydrolysis and fractionation revealed for the first time specific protein/peptide fractions identified from CM as potential antidiabetic agents with well-characterized mechanisms of action at the cellular and molecular levels.

This opens interesting opportunities for the application of CM-derived peptides as therapeutic tools in diabetes and its related disorders. Bioactive natural or synthetic peptides and their applications in drug discovery in different human diseases have gained interest over time [16,17]. However, promising application opportunities of CM-derived agents in frug discovery and clinic depend on the identification of the exact bioactive components contained in CM protein hydrolysates and our understating of their mode of action at both cellular and molecular levels. Moreover, this requires a rational validation and characterization of the different peptides identified in very heterogeneous CM whey protein hydrolysates using similar settings for the study of bioactivity in cells. Moreover, the *in vivo* validation of the peptides in appropriate diabetic models would be required for possible development of potent CM-derived peptides and their safe clinical application in diabetes.

This challenging context defines the main aim of our research study, which is to consolidate our previous findings on crude CM proteins and hydrolysates and their effects on different molecular pathways, such as DPP-IV and IR. Thus, we believe the next step would be the functional profiling and validation of the bioactivity of pure and synthetic peptides derived from CM whey protein hydrolysates. Indeed, we synthesized peptides based on the peptide sequencing performed on the heterogenous CM whey hydrolysates that we previously reported [9,12,14]. The aim of the study was to functionally validate these CM-derived peptides and determine if they still show any bioactivity using settings similar to those previously applied to the crude CM whey protein hydrolysates. The study also aims to further understand the molecular mechanism of the antidiabetic properties of CM and ultimately identify the peptides that may have therapeutic applications in diabetes based on their pharmacological effects on IR activity and glucose uptake. For this, we examined the functional effects of the synthesized CM-derived peptides previously identified from crude and heterogeneous hydrolysates on IR activity, AKT phosphorylation, and glucose uptake in HEK293 and HepG2 cell lines. We also investigated the possibilities of peptide binding to IR using an *in silico* approach based on the crystallographic structure of insulin-bound IR. Thus, the novelty of the study resides in the dual impact in the field of the antidiabetic properties of CM by further characterizing the molecular pathways involved and validating specific CM-derived peptides with well-known sequences and profiles *in vitro* and *in silico*.

## Materials and methods

### Chemicals and reagents

#### Peptides.

Nine peptides were selected from synthesis based on our previous study on exploration of antidiabetic peptides via inhibition of DPP-IV enzyme and potentiation of IR activity [9]. Peptides were mainly selected from the crude pepsin digestion-derived hydrolysates based on the results of their *in silico* binding affinity to DPP-IV enzyme and *in vitro* activation of IR and its canonical intracellular kinases observed in both HEK293 and HepG2 cells [9]. All peptides were synthesized from GenScript (Piscataway, NJ, USA) with ≥ 95% purity, shipped lyophilized, and dissolved to 100 mg/ml in water (Table 1).

**Table 1:** List of the peptides used in the study with their sequence and molecular weight (M.W).

Peptides	Sequences	M.W. (Da)
P1	LRPFL	716.9
P2	PAVACCLPPLPCHM	1686.1
P3	LPVPQ	624.7
P4	MPVQA	616.6
P5	WK	350.4
P6	LPVP	478.5
P7	VPF	397.4
P8	VPV	349.3
P9	YPI	427.5

#### Plasmids and reagents.

The mammalian expression plasmids coding for the human IR-Rluc and its signaling protein substrate, IRS1-YFP, were generously provided by Dr. Tarik Issad (Cochin Institute, Paris, France). Human insulin was purchased from Sigma Aldrich (St. Louis, MO, USA). The antibodies recognizing IR and AKT (the native and the phosphorylated forms) were from Cell Signaling (Boston, MA, USA); Coelenterazine h for BRET assays and Glucose Uptake-Glo kit were purchased from Promega (Madison, WI, USA); the bicinchoninic acid (BCA) protein assay and Lipofectamine 2000 for cell transfection were purchased from ThermoFisher Scientific (Waltham, MA, USA). 96-white plates were from Greiner Bio-One (Frickenhausen, Germany).

### *In vitro* experiments

#### Cell culture and transfection.

HepG2 and HEK293 cells were maintained at 37°C, 5% CO2 in complete RPMI and Dulbecco’s modified Eagle’s medium (DMEM), respectively, containing 0.3 mg/ml glutamine, 100 IU/ml penicillin, and 100 µg/ml streptomycin) supplemented with 10% fetal calf serum (Gibco, Carlsbad, CA, USA). The transient transfection in HEK293 cells was carried out in 75 cm flask containing 10^7^ cells using Lipofectamine 2000 and 5 µg of IR-Rluc and 5 µg of IRS1-YFP (for BRET and phosphorylation experiments) as previously described [8–10]. All assays were performed 48 hours post-transfection.

#### Protein phosphorylation assay.

HepG2 cells were seeded onto 6-well plates at 10^6^ cells/well cells and starved overnight in DMEM serum-free media before their treatment with either insulin (1 μM) or peptides (0.1 mg/ml) for 10 minutes at 37°C. Following treatment, cells were then lysed with RIPA lysis buffer and thereafter centrifuged at 12000 × g for 30 minutes. Thereafter, the extracted cell lysate proteins were quantified using the bicinchoninic acid (BCA) protein assay. To examine the phosphorylation status of IR and AKT, 30-40 μg of proteins were loaded on 10% SDS-PAGE gel followed by western blot using the following antibodies: the mouse monoclonal anti-phospho-IR (pIR-Tyr1334)(1:1000 dilution) and the mouse monoclonal anti-phospho-AKT (pAKT-S473)(1:2000 dilution) as previously described [9,10]. The corresponding total proteins loaded were also analyzed in parallel using the specific antibodies against the native forms of the different proteins. Densitometry analysis of the different western blots was performed using Image Studio Version 5.2 software from LICOR (Lincoln, Nebraska, USA).

#### BRET assay.

BRET technology was used for IR activation and its interaction with IRS1 as previously described [9,10]. Cells transiently co-expressing IR-Rluc and IRS1-YFP were first seeded in 96-well white plates and used for dose-response analysis and endpoint single or combined treatments with insulin and peptides. For this, cells were first starved overnight in DMEM serum-free media, washed with 50 μl/well of PBS and treated for 60 minutes at 37°C with 40 μl of PBS with or without the indicated doses of either insulin or the different peptides alone or in combination. Following treatments, 10 μl of Coelenterazine h was added to a final concentration of 5 µ M and BRET measurements were carried out using the Tristar 2 multi-label plate reader (Berthold, Germany), allowing luminescence recording and signal integration at 480 nm and 540 nm.

#### Glucose uptake assay.

Glucose uptake in HepG2 cells was carried out using Glucose Uptake-Glo Assay (Promega). For this, cells seeded in a 96-well white plate (50,000 cells/well) were first washed with PBS and starved in phenol red-, glucose-, and serum-free medium (Dulbecco’s modified Eagle’s medium containing 0.01 mM HEPES, 0.3 mg/ml glutamine, 100 IU/ml penicillin, and 100 µg/ml streptomycin). Then, cells were first treated or not with either insulin or peptides for 60 minutes at 37°C. The treatment was removed, and the cells were washed once with PBS and then incubated with 50 μL/well of freshly prepared 2-deoxyglucose (500 μM) for 10 minutes at room temperature. The uptake process was stopped and neutralized, followed by the addition of 50 μl/well of 2-deoxyglucose-6-phosphate (2DG6P) detection reagent and incubation for 15-60 minutes at room temperature. The glucose-6-phosphate dehydrogenase (G6PDH) oxidizes 2DG6P to 6-phosphodeoxygluconate (6PDG) and reduces NADP + to NADPH. The reductase uses NADPH to convert the proluciferin to luciferin, which is then used by Ultra-Glo™ recombinant luciferase to produce luminescence. This luminescence, proportional to the amount of 2-deoxyglucose transported inside the cells upon treatment, was then measured using a 0.3–1 second integration on GloMax^®^ Discover luminometer according to Glucose Uptake-Glo™ protocol.

### *In silico* experiments

#### Protein structure preparation.

The crystal structure of the human insulin ectodomain (PDB ID: 6CEB) obtained from the Protein Data Bank (PDB) [18] was prepared using the Protein Preparation Wizard of Schrödinger Suite 2022-4 [19]. The protein underwent preprocessing steps, including hydrogen addition, removal of unwanted water molecules, proper bond order assignment, adjustment of ionization states, orientation of disoriented groups, addition of disulfide bonds, partial charge assignment, and correction of residues with missing atoms and side chains. The unwanted ligands and chains were removed, and tautomeric states were generated at pH 7.0. Finally, the protein structure was optimized and minimized using the OPLS 2005 force field to maintain geometric and structural stability [20].

#### Binding site identification and grid generation.

To identify potential binding sites, we explored the protein structure using Schrödinger SiteMap [21]. SiteMap identifies and ranks possible binding sites based on various descriptors, providing a comprehensive evaluation of the physiochemical properties of each site. SiteMap generates a draggability score (Dscore), which characterizes each binding site in terms of its size, solvent exposure, strength of interactions between site points and the receptor, hydrophobic-hydrophilic balance, and the extent of hydrogen bond donation and acceptance [22,23]. The receptor grid was generated by selecting the co-crystallized ligand to perform site-specific docking. OPLS 2001 force field and the default parameters were used with the van der Waals radii of the receptor atom were scaled to 1.0, and the partial charge cutoff value was set to 0.25.

#### Peptide docking.

Peptide docking was conducted to determine the most probable binding orientation of the peptides with the insulin receptor, analyze interfacial molecular interactions, and estimate binding free energy. Standard precision (SP) flexible docking was carried out using Schrödinger Glide version 2022-2 with default settings [24]. Peptides were docked using the default parameters, and the resulting poses were ranked according to the GlideScore (GScore) scoring function [25]. Poses with the lowest GScore values were selected for further analysis.

#### Binding free energy calculation.

Following docking, the top-ranked poses were analyzed to assess various types of interactions, including hydrogen bonds, hydrophobic interactions, salt bridges, π–π, and π-cation interactions [26]. These best-docked poses were then evaluated for binding free energy using molecular mechanics generalized Born surface area (MM-GBSA) in an implicit solvent model. The MM-GBSA binding energy was calculated with Schrödinger Prime, utilizing the OPLS 2005 force field and the VSGB 2.0 implicit solvent model [27,28].

### Data presentation and statistical analysis

For BRET, the data were normalized by taking as 100% of the maximal insulin-induced BRET signal in the vehicle and analyzed using GraphPad Prism software (San Diego, CA, USA). Statistical analyses were performed statistical significance between the different treatments and conditions as indicated in the figures and tables and their captions. For this, the ordinary one-way ANOVA was used to determine the statistical significance of the treatments with the different peptides or insulin (single or increasing doses) compared to the untreated condition. The significance is indicated in the figures by stars (****p-value <  0.0001, ***p-value <  0.001, ** p-value <  0.01, *  p-value <  0.05), and the absence of stars means non-significant (*p-value >  0.05*). However, the two-way ANOVA was used for multiple comparisons to determine the statistical significance between the different peptides when compared to each other in the different assays as described in the tables. In this case, the different letters indicate statistically significant effects (*p-value <  0.05*), while the same letters indicate no significant difference between the different treatments compared to each other.

## Results and discussion

### Effects of synthetic CM-derived peptides on IR activity

In the previous study, we identified bioactive trypsin-/pepsin-digested CM protein hydrolysates inhibiting DPP-IV, positively modulating IR and kinase activity, and promoting glucose uptake in HEK293 and HepG2 cells [9,12,14]. This can be considered as one of the putative molecular basis for the antidiabetic properties of CM supported by many *in vitro* and *in vivo* studies [1–4]. The CM protein hydrolysates were characterized by their heterogeneous content of peptides with different lengths that could be the bioactive peptides in CM hydrolysates on DPP-IV and IR activity and glucose transport [9,12,14]. At this stage, it is not clear if such a positive effect of CM-derived hydrolysates was due to the mixture of peptides or specific bioactive peptides targeting the different DPP-IV and IR signaling pathways. The analysis and the sequencing of the different CM fractions led us to identify nine potential peptides we chemically synthesized and resuspended in water (Table 1). Thus, in this study, we aimed to examine the bioactivity of these CM-derived peptides using similar cellular models and functional approaches, as previously reported, by examining their action on IR activity by BRET technique in live HEK293 cells as well as kinase phosphorylation and glucose uptake in HepG2 cells.

**Fig 1:**
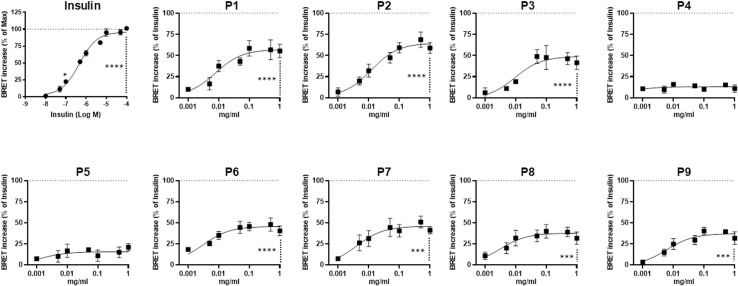
Dose-response BRET analysis of the synthetic CM-derived peptides. HEK293 cells transiently co-expressing IR-Rluc and IRS1-YFP were treated for 1 hour at 37°C with increasing insulin doses (in Log M) or the peptides (in mg/ml). Cells were then used for BRET measurements as described in Materials and Methods. Data are normalized as % of the maximal response of insulin (first panel) and plotted as the mean ± SEM of four independent experiments performed in triplicate. The stars indicate the statistical significance of the BRET signals measured in cells treated with increasing doses of insulin or peptide compared to those measured in untreated cells. The absence of stars with P4 and P5 means a non-significant difference (*p-value >  0.05*).

The pure and synthetic peptides were first profiled and validated for their bioactivity on IR in live HEK293 cells using the bioluminescence resonance energy transfer (BRET) technique, which measures the activation of IR in live cells as the increase in the physical proximity between IR-Rluc (BRET donor) and YFP-tagged insulin receptor substrate-1 (IRS1-YFP)(BRET acceptor). For this, dose-response experiments were performed in HEK293 cells treated for 60 minutes with increasing doses of insulin or peptides before BRET measurements. As shown in Fig 1, the insulin used as a positive control induced a dose-dependent BRET increase, and in parallel, the peptides, except P4 and P5, also promoted a partial but significant BRET increase that occurred in a dose-dependent manner. The curves showed different efficacies among the peptides, with the plateau reached at ~ 0.1 mg/ml of peptides (Fig 1). Overall, the peptide efficacy (E_max_) varied between 13% to 65%, and their potency (EC_50_) ranged from 3 μg/ml to 12 μg/ml, as summarized in Table 2. Indeed, P2 appears to be the most efficient peptide, followed by P1/P3/P6/P7 and then P8/P9 with lower responses to IR activity (Fig 1 and Table 2). This demonstrates the bioactivity of the selected synthetic peptides on IR function in HEK293 cells, consistent with our previous studies on the crude CM whey proteins and their hydrolysates showing a positive effect on BRET between IR-Rluc and IRS1-YFP [9].

**Table 2:** The effect of the peptides on BRET responses in HEK293 cells normalized as a percentage (%) of insulin’s response taken as 100%. Maximal BRET increase and EC_50_ values are the mean ± SD of four independent experiments performed in triplicate. *The different letters indicate statistically significant effects (p-value <  0.05), while the same letters indicate no significant difference, between the different treatments compared to each other.*

Peptides	% of maximal BRET	EC_50_ (μg/ml)
Insulin	100^a^	0.46 ± 0.06 (μM)^e^
P1	57 ± 5^b^	7.97 ± 4.94^f^
P2	65 ± 4^b^	12.30 ± 5.66^f^
P3	49 ± 5^b,d^	9.44 ± 7.48^f^
P4	13 ± 1^c^	ND
P5	15 ± 3^c^	ND
P6	46 ± 3^b,d^	3.13 ± 1.72^f^
P7	46 ± 4^b,d^	4.12 ± 2.78^f^
P8	38 ± 4^b^	3.15 ± 2.21^f^
P9	37 ± 3^b^	6.11 ± 3.05^f^

In this set of single-treatment and dose-response BRET experiments in HEK293 cells, our data demonstrate the partial positive action of most peptides, except P4 and P5, on IR activity. The peptides partially increased, in a dose-dependent manner and to a different extent, the BRET signals compared to insulin, with P2 being the most efficient (65%) (Fig 1 and Table 2). Moreover, the peptides showed similar potency within μg/ml (μM) range. The agonistic action of the peptide on IR was observed despite differences in peptide lengths and sequences, consistent with our previous observations on intact CM whey proteins as well as their pepsin-generated hydrolysates [9]. Indeed, these observations indicate that the peptides previously identified as a mixture in crude CM whey hydrolysates are also bioactive on IR activity in HEK293 cells when used individually as pure peptides. This further consolidates our previous peptide analysis, identification, and selection. However, this does not indicate whether all the peptides directly bind IR and promote or not its downstream signaling.

**Fig 2:**
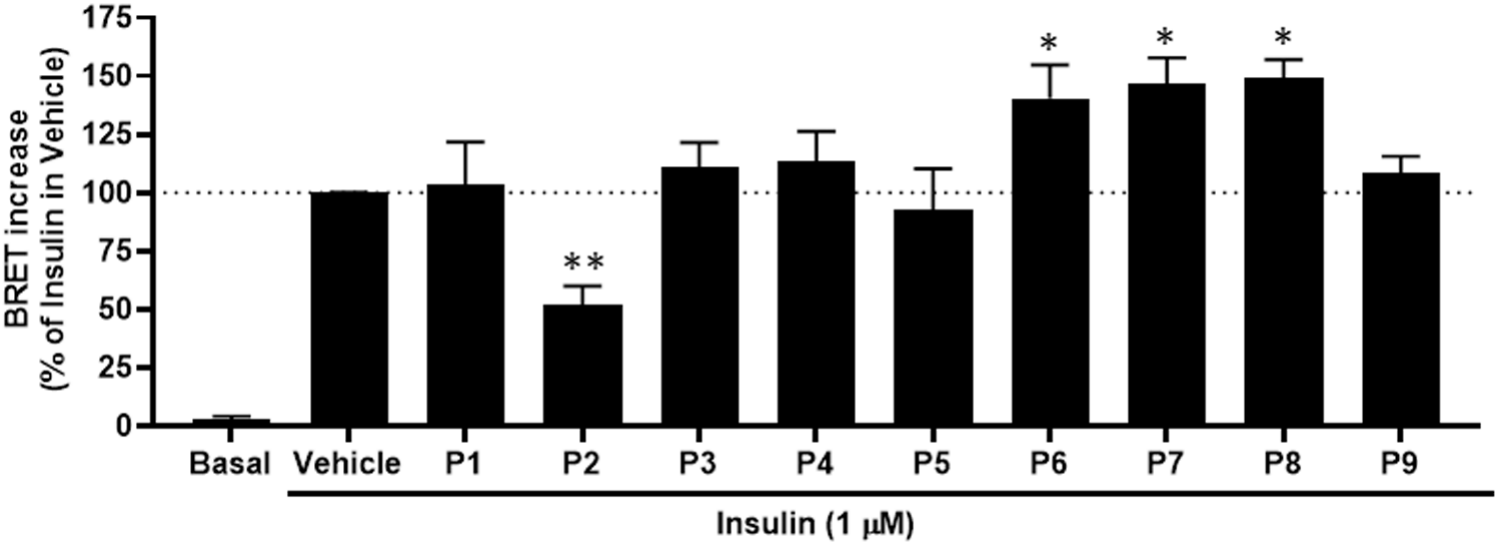
The effect of the synthetic CM-derived peptides on insulin-mediated BRET responses. HEK293 cells transiently co-expressing IR-Rluc and IRS1-YFP were treated or not (Basal) for 1 hour at 37°C with 1 μM of insulin in the absence (Vehicle) or presence of 0.1 mg/ml of the peptides. Cells were then used for BRET measurements as described in Materials and Methods. Data are normalized as % of insulin’s response in the vehicle and represented as the mean ± SEM of five to seven independent experiments performed in triplicate. The stars indicate the statistical significance of the BRET signals measured in cells treated with the combination of insulin and peptides compared to those measured in cells treated with insulin alone (Vehicle). The BRET signals measured in all the treatments were statistically significant compared to those measured in untreated cells (Basal) (stars not shown).

Another interesting aspect of our previous studies on CM whey proteins and hydrolysates was their positive allosteric/synergistic action on IR when combined with insulin [9]. This suggests that such CM protein fractions directly or indirectly induce and/or stabilize IR in a more favorable conformation different from the one induced by insulin alone. In this context, we profiled the peptides at a saturating dose (0.1 mg/ml) in combination with a sub-saturating does (1 μM) of insulin in BRET assay in HEK293 cells (Fig 2). As a result, when compared to insulin treatment alone (Vehicle), we obtained three different profiles among the nine peptides: (i) non-efficient peptides (P1, P3, P4, P5, and P9), (ii) an antagonistic peptide (P2) that significantly decreased by ~ 50% the insulin-dependent BRET response, and (iii) potentiating peptides (P6, P7 and P8) that significantly increased the insulin-dependent BRET response beyond 100% (up to147%) (Fig 2 and Table 3).

**Table 3:** The effect of the peptides on insulin-induced BRET responses in HEK293 cells normalized as a percentage (%) of insulin’s response in the vehicle taken as 100%. Maximal BRET increase values are the mean ± SD of five to seven independent experiments performed in triplicate. *The different letters indicate statistically significant effects (p-value <  0.05), while the same letters indicate no significant difference, between the different treatments compared to each other.*

Peptides	% of maximal BRET
Insulin	100^a^
P1	104 ± 18^a^
P2	52 ± 8^b^
P3	111 ± 11^a^
P4	114 ± 13^a^
P5	92 ± 18^a,c^
P6	135 ± 13^d^
P7	147 ± 11^d^
P8	121 ± 14^d^
P9	109 ± 7^a^

The combined treatments (insulin +  peptides) revealed the potentiating effect of three peptides (P6, P7, and P8) on insulin-promoted IR activity, consistent with our previous study on intact CM whey proteins and their hydrolysates [9]. This suggests that these three peptides may be behind the potentiating action of CM hydrolysates, through their allosteric (direct or indirect) binding to IR. P6, P7, and P8 were found in our trypsin-hydrolyzed CM whey proteins, presenting some similarities in size and sequence and sharing the common dipeptide VP that may explain the results (Table 1). This may provide an interesting structure-activity relationship explanation and a rationale for any future development of more potent and efficient peptides based on these three peptides. Interestingly, P2 decreased insulin-mediated BRET signal, suggesting an antagonistic action (Fig 2 and Table 3). This peptide was previously found in pepsin-hydrolyzed CM whey fraction 5 (H5) [9]. The negative action of P2 on insulin’s BRET response is inconsistent with its positive effect when applied alone (Fig 1), suggesting that P2 has a dual action on IR, an agonistic action when applied alone, and an antagonistic effect when combined with insulin. P2 may negatively interfere with insulin binding on IR and/or stabilize a less active IR conformation than insulin alone, leading to a lower BRET response. Studies showed that some peptides exhibit mixed agonistic and antagonistic properties affecting IR activation and signaling [29]. Thus, P2 could plausibly also have such mixed effects, making its differential effect with and without insulin co-treatment conceivable. In addition, the inhibitory effect of P2 may also be due to its size (14 residues) being the longest peptide tested in the study, with the presence of proline, alanine, and methionine at the C-terminal reported to have inhibitory properties [30], which may involve either a direct or indirect effect on insulin binding on IR. For instance, P2 may simply bind to a site that creates a steric hindrance preventing insulin from binding to IR due to the peptide’s size.

The other non-effective peptides (P1, P4, P5, and P9) in the combined with no significant effect on insulin-induced BRET responses are contrasted in their length and sequence. However, P1 and P9, but not P4 and P5, were partially active in the BRET assay when applied alone (Fig 1). The absence of effect with P5 and P9, when combined with insulin, may be due to their shortness and the absence of the dipeptide VP found in the efficient peptides P6, P7, and P8. For P1 and P4, both are longer (pentapeptides) than P5 and P9, with completely different sequences but a similar functional profile on IR activation (but also AKT phosphorylation and glucose uptake). Moreover, P1 and P4 showed better scores for binding to IR, as discussed below, which does not seem to be consistent with allosteric modulation of IR.

### Effects of synthetic CM-derived peptides on protein phosphorylation

**Fig 3:**
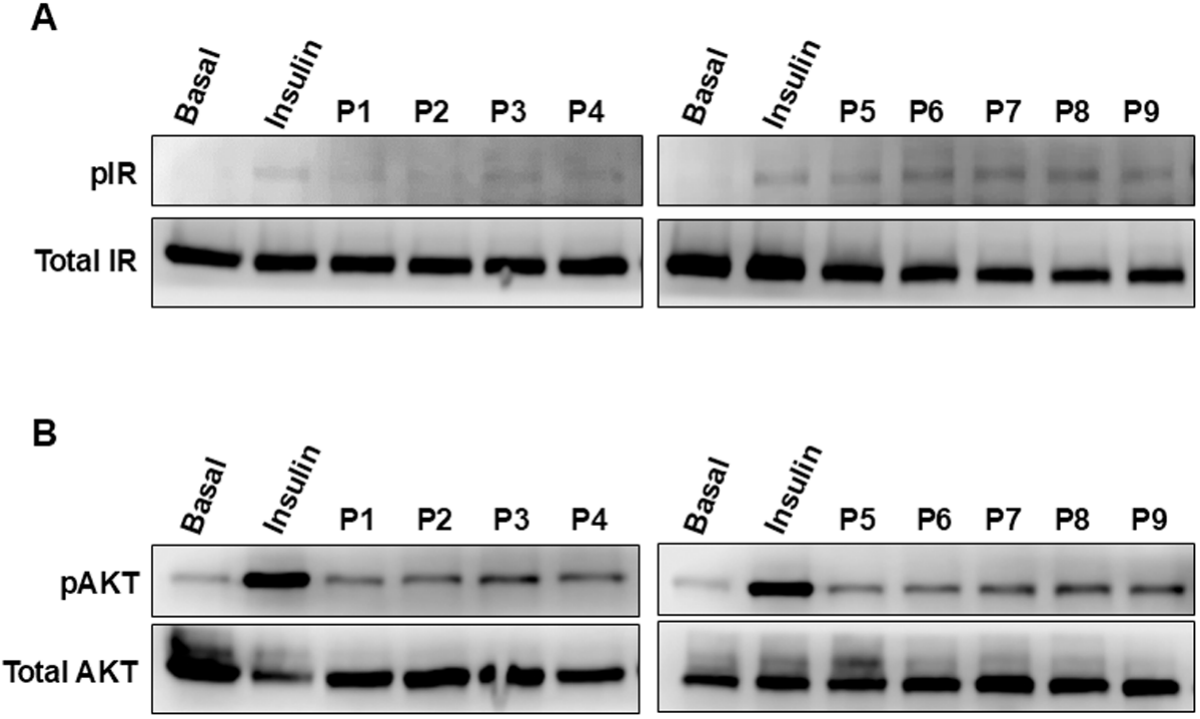
The effect of the synthetic CM-derived peptides on IR and AKT phosphorylation in HepG2 cells. HepG2 cells endogenously expressing IR were treated or not (Basal) for 10 minutes at 37°C with 1 μM of insulin or 0.1 mg/ml of the peptides. After cell lysis and protein extraction, the level of IR (**A**) and AKT (**B**) proteins and their respective phosphorylation were assessed by SDS-PAGE followed by western blot. The blots are representative of 3 independent experiments.

To further characterize the bioactivity of the synthetic CM-derived peptides in our cellular model, we examined their effects on the phosphorylation of IR and its downstream signaling kinase, AKT, in HepG2 cells. The activation of insulin-induced IR is known to promote an early event characterized by receptor phosphorylation, followed by the downstream activation of PI3-kinase pathway leading to AKT phosphorylation and its activation [31]. AKT activation controls the plasma membrane translocation of the glucose transporter GLUT4 [31]. As shown in Fig 3A, all the peptides applied at 0.1 mg/ml led to a moderate induction of IR phosphorylation, which is comparable to insulin-mediated response. Similarly, all the peptides induced a significant AKT phosphorylation that was however partial when compared to insulin’s response (Fig 3B).

Together, these phosphorylation data suggest that the peptides have partial positive effects on IR and AKT phosphorylation in HepG2 cells. This is consistent with dose-response BRET data in HEK293 cells shown in Fig 1, indicating that the peptides induce IR activation resulting in receptor and AKT phosphorylation. However, P4 and P5 applied alone did not promote any significant BRET increase (Fig 1), while they both led to IR and AKT phosphorylation. The cell line background, HEK293 versus HepG2, or the assay sensitivity, may explain such a difference. Alternatively, P4 and P5 may induce/stabilize an IR conformation prone to receptor phosphorylation but not BRET changes. Indeed, BRET changes may reflect receptor activation and conformational changes, leading to phosphorylation. Thus, P4 and P5 binding may result in specific changes of IR conformation that could not be detected by BRET but led to receptor phosphorylation.

**Fig 4:**
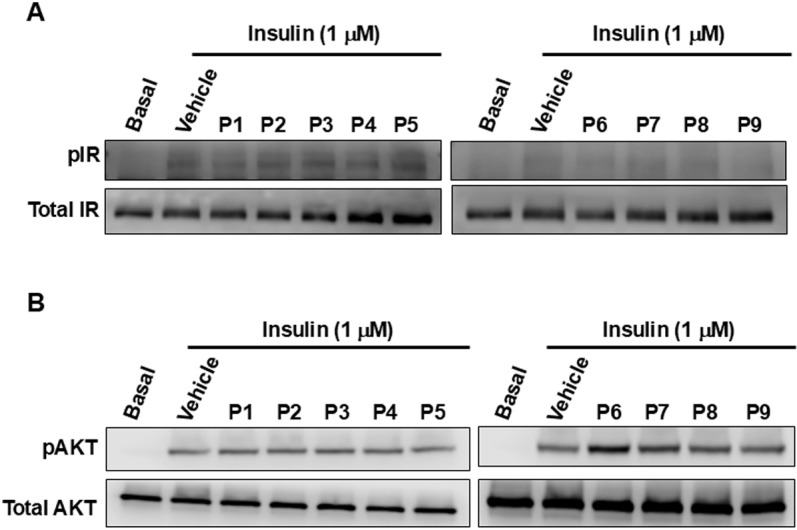
The effect of the synthetic CM-derived peptides on insulin-induced IR and AKT phosphorylation in HepG2 cells. HepG2 cells were treated or not (Basal) for 10 minutes at 37°C with 1 μM of insulin in the absence (Vehicle) or presence of 0.1 mg/ml of the peptides. After cell lysis and protein extraction, the level of IR (**A**) and AKT (**B**) proteins and their respective phosphorylation were assessed by SDS-PAGE followed by western blot. The blots are representative of 3 independent experiments.

In combination treatment, none of the peptides significantly affected insulin-mediated IR phosphorylation (Fig 4A). However, for AKT phosphorylation, the peptides P6 and P7 slightly potentiated the insulin-mediated response compared to insulin alone (Fig 4B).

The data on AKT phosphorylation are consistent with our BRET data in HEK293 cells, which showed that P6 and P7 (also P8) potentiated insulin-induced BRET increase (Fig 2). By contrast, the absence of any potentiation of insulin-induced IR phosphorylation in HepG2 cells by all the peptides is indeed not correlated with the BRET data showing P6, P7, and P8 having a significant potentiating effect on insulin-promoted BRET increase, as shown in Fig 2. Moreover, P2 has no inhibitory action on IR phosphorylation induced by insulin, which is inconsistent with the BRET observations in HEK293 cells (Fig 2). More importantly, the absence of a synergistic effect of the peptides on IR phosphorylation is not consistent with our previous observations with CM whey proteins and their hydrolysates [9]. Again, the cell line background, HEK293 versus HepG2, or the assay sensitivity, may explain such a difference. We can also speculate that the peptides combined with insulin may induce/stabilize IR conformational changes prone to BRET but not phosphorylation changes.

### Effects of synthetic CM-derived peptides on glucose uptake

**Fig 5:**
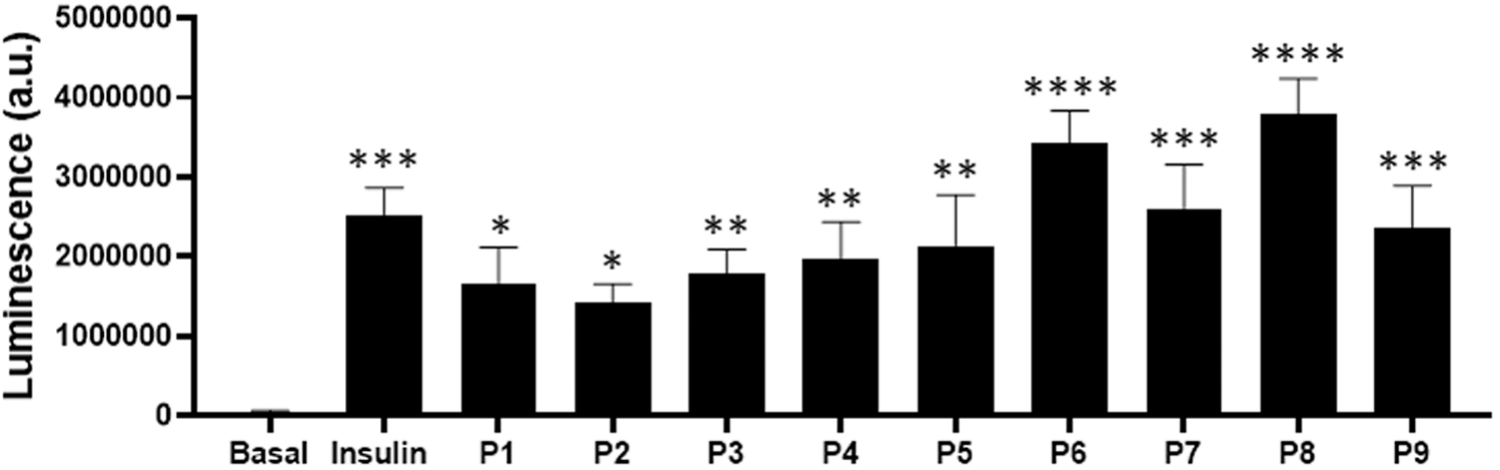
Effect of the synthetic CM-derived peptides on glucose uptake in HepG2 cells. HepG2 cells were treated or not (Basal) for 1 hour at 37°C with 1 μM of insulin or 0.1 mg/ml of the peptides, and glucose uptake was then measured in live cells. The specific luminescence signals are represented as the mean ± SEM of five independent experiments performed in triplicate. The stars indicate the statistical significance of the signals measured in cells treated with insulin or peptides compared to those measured in untreated cells (Basal).

Together BRET and phosphorylation data demonstrate the bioactivity of CM-derived peptides in HEK293 and HepG2 cells in terms of IR activity and kinase signaling. To assess the bioactivity of the peptides in a more integrated cell response, we also examined their effects on glucose uptake in HepG2 cells. As shown in Fig 5, all peptides (0.1 mg/ml) significantly induced glucose uptake by HepG2 cells to different extent. Most of them showed partial action ( ~ 56 – 84%) when compared with insulin (1 μM); however, while P7 and P9 lead to a similar response as insulin, P6 and P8 promoted a higher response ( ~ 135 – 150%) when compared with all other treatments, including insulin (Fig 5) (Table 4).

**Table 4:** The effect of the peptides on glucose uptake in HepG2 cells normalized as a percentage (%) of insulin’s response taken as 100%. Data are the mean ± SD of five independent experiments performed in triplicate. *The different letters indicate statistically significant effects (p-value <  0.05), while the same letters indicate no significant difference, between the different treatments compared to each other.*

Peptides	% of glucose uptake
Insulin	100^a^
P1	66 ± 18^b^
P2	56 ± 9^b^
P3	71 ± 12^b^
P4	78 ± 18^b^
P5	85 ± 25^b,c^
P6	136 ± 16^d^
P7	103 ± 22^a^
P8	150 ± 18^e^
P9	94 ± 21^c^

These data confirm the functional activity of all the peptides on glucose transport in HepG2 cells with different efficacies. Also, this further suggests that P6, P7, and P8 are more potent and efficient in our model, consistent with IR and AKT phosphorylation data in HepG2 cells. Indeed, in glucose transport, the peptides had similar efficiency as insulin, except P6 and P8, which significantly showed a higher response. The correlation between AKT phosphorylation and glucose uptake is consistent with the key role of AKT activation in controlling membrane glucose transporter (GLUT4) translocation and function [31]. These observations suggest the role of the peptides in the effect of the crude CM protein hydrolysates on glucose uptake in HepG2 cells, as previously reported [9]. Interestingly, even the short peptides, including the dipeptide P5 and the tripeptides such as P7, P8, and P9, were functional in BRET (except P5), phosphorylation, and glucose uptake. The bioactivity of very short peptides (dipeptides) was reported in many situations, including in glucose metabolism and diabetes [32–36].

### Molecular docking of the binding of the synthetic CM-derived peptides on insulin-bound IR

Our functional *in vitro* data demonstrate the bioactivity of the different synthetic CM-derived peptides via targeting IR, AKT, and glucose uptake. However, this do not provide any evidence for their direct binding to IR. To address this point, we used an *in silico* approach based on molecular docking. For this, we considered the situation of combined treatments *in vitro* in HEK293 and HepG2 cell lines showing a positive allosteric/synergistic action of CM protein fractions when combined with insulin [9]. Thus, for our molecular docking, we used the 3D structure of the ectodomain of IR bound with insulin (PDB ID: 6CEB) obtained from the Protein Data Bank (PDB) and solved by single-particle cryo-electron microscopy [18]. Five possible binding sites in the IR ectodomain were identified using Schrodinger SiteMap. SiteMap computes descriptors like Dscore to provide further insight into the physiochemical properties of a binding site. Among the five possible binding sites, site 2 in IR had a high Dscore, indicating a favorable binding environment regarding spatial and physicochemical properties, which generally reflects binding affinity potential. Site 2 is located adjacent to the insulin binding site [18]. To facilitate the docking analysis, a receptor grid was generated specifically for site 2 using the receptor grid generation tool of Schrodinger Maestro. The peptides were further docked into site 2 to explore potential interactions, binding affinities, and orientations within this specific binding site.

Based on the docking score (GlideScore or GScore) and molecular mechanics-generalized Born surface area (MMGBSA) calculation used for binding free energy, P1 and P4 exhibited better scores (Table 5). Indeed, P1 and P4 present the best combination of GScore (-6.30 and -6.03 kcal/mol, respectively) and binding free energy (-64.16 and -69.02 kcal/mol, respectively) (Table 5). However, P3, P5, and P9 also showed good GScore but higher binding free energy. For P2, P6, P7, and P8, the scores were weaker with P2 and P6 having the weakest binder peptides when considering either GScore or binding free energy, respectively (Table 5).

**Table 5:** Binding scores of the synthetic CM-derived peptides at site 2 of IR determined by molecular docking on insulin-bound IR.

Peptides	GScore (kcal/mol)	MM-GBSA binding free energy (kcal/mol)
P1	-6.30	-64.16
P2	-3.71	-52.88
P3	-6.22	-44.26
P4	-6.03	-69.02
P5	-7.58	-45.68
P6	-5.07	-26.08
P7	-5.76	-49.36
P8	-5.66	-47.67
P9	-6.20	-54.77

**Fig 6:**
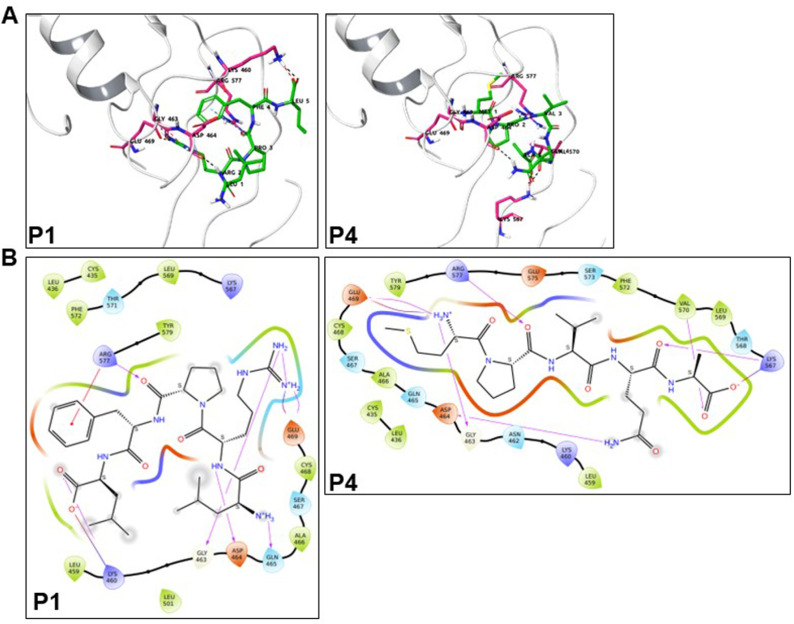
The interaction of P1 and P4 with IR in the molecular docking analysis. **A**. Ribbon diagram showing the hydrogen bonds (black dotted lines), salt bridge (red dotted lines), and pi-cation (cyan dotted lines). **B.** Interactions between the different residues of P1 and P4 and IR, including the hydrogen bonds (pink arrows), π-π interactions (green lines), π-cation (red lines), salt bridges (red-blue line), and the hydrophobic interacting residues (green color).

Therefore, we focused on P1 and P4 for further binding analysis, and we docked the putative poses of these two peptides within binding site 2 in IR. The analysis showed the different interacting residues involved in P1, P4, and IR, as illustrated in Fig 6.

The docking analysis established a possible correlation between our *in vitro* pharmacological and functional analysis of the peptides and their binding on IR *in silico*. Our BRET results indicated three different profiles of peptides: non-efficient peptides (P1, P3, P4, P5, and P9), an antagonistic peptide (P2), and potentiating peptides (P6, P7, and P8). The three potentiating peptides present share the common dipeptide VP that may explain the results, but surprisingly, they did not show high scores in our molecular docking analysis (Table 5) and did not seem to be the best binders in site 2 of the insulin-bound IR ectodomain. For P6, the analysis showed even an unfavorable situation with a higher binding free energy (-26.08 kcal/mol). The BRET data with P6 are consistent with those on AKT phosphorylation being the unique peptide potentiating the response when combined with insulin. By contrast, P3 also looks very related to the three peptides P6, P7, and P8, having VP dipeptide, with similar GScore and binding free energy in our molecular docking analysis (Table 5), but it did not affect insulin-mediated response. Indeed, P3 (LPVPQ) looks very close to P6 (LPVP), and the presence of proline in peptides activates IR, suggesting that the presence of the polar glutamine (Q) residue at the C-terminus is detrimental to the bioactivity of the peptide when combined with insulin [37]. On the other hand, the negative effect of P2, previously found in pepsin-hydrolyzed CM whey fraction 5 (H5) [9], on insulin’s BRET response (Fig 2) is unexpected and contrasted with its positive effect when applied alone (Fig 1), as discussed above. Our molecular docking analysis showed that P2 was the weakest binder peptide in site 2 of the insulin-bound IR ectodomain, suggesting that P2 may bind elsewhere, including the orthosteric binding site of insulin, which would lead to a competitive/inhibitory effect. Surprisingly, P1 and P4 showed the best scores in the molecular docking analysis for their binding to the insulin-bound IR ectodomain (Table 5). This may be due to their size and hydrophobic profile involving various hydrophobic interactions with key residues in site 2 of IR ectodomain (Fig 6). Finally, the weak binding score in S2 site of IR with the peptides does not completely rule out their binding and the existence of other allosteric binding sites needs to be investigated both *in silico* and experimentally.

## Conclusion

We previously identified a mixture of peptides from CM protein hydrolysates with antidiabetic properties and validated their bioactivity on IR function, downstream kinase signaling, and glucose uptake. This study aimed to synthesize and validate selected peptides using similar experimental settings. Overall, the data confirm the bioactivity of CM-derived peptides on IR activity and its downstream signaling and on glucose uptake in cell lines. Moreover, our *in silico* part provides a speculative analysis of the putative interaction and binding of the peptides with insulin-bound IR. The correlative and comparative analysis of the different parameters and assays used in the study shows some discrepancies in the heterogenic profiles of the peptides (Table 6).

**Table 6:** Overall correlation between the synthetic CM-derived peptides in the different experimental parameters and assays. The gray color indicates the absence of a significant effect, while green and red indicate either an agonistic or an antagonistic effect, respectively.

Assays	P1	P2	P3	P4	P5	P6	P7	P8	P9
BRET in single treatment									
BRET in combined treatment									
IR phosphorylation in single treatment									
IR phosphorylation in combined treatment									
AKT phosphorylation in single treatment									
AKT phosphorylation in combined treatment									
Glucose uptake									
*In silico* analysis									

It’s clear that all the peptides showed an agonistic action in BRET phosphorylation and glucose uptake, expect P4 and P5, lacking a significant effect in BRET. This demonstrates the bioactivity of the peptides and illustrates a good correlation between the different parameters and functional assays. However, such an agonistic effect of the peptides does not seem to be correlated with their binding to the allosteric S2 site in IR, with only P1 and P4 showing a good score. The existence of other potential binding sites in IR is plausible. Also, such absence of binding for most of the peptides may be correlated with our data in the combined treatments with insulin, which shows that none of the peptides had any significant effect on insulin-mediated IR phosphorylation. However, this also illustrates remarkable discrepancies among the peptides and the parameters when compared to each other. Indeed, our best *in silico* peptide binders, P1 and P4, had no significant potentiating action in any assay. In contrast, the weak binders, P6 and P7, significantly potentiated BRET in HEK293 cells and AKT phosphorylation in HepG2 cells (Table 6), which again suggests the existence of other potential allosteric binding sites in the receptor to be determined. Yet, these differential profiles among the selected peptides in the different assays may explain their mixed and cumulative effects when they are all together in the crude and CM hydrolysates previously reported [9].

Nevertheless, the novelty of the study resides in the dual impact in the field of the antidiabetic properties of CM by further characterizing the molecular pathways involved and validating specific CM-derived peptides with well-known sequences and profiles *in vitro* and *in silico*. Moreover, these findings highlight the therapeutic potential of CM-derived peptides in enhancing insulin sensitivity, IR signaling, and glucose transport. However, further *in vitro* and *in silico* binding and functional analysis are required for a more comprehensive structure-activity relationship study. Moreover, *in vivo* validation of the peptides in appropriate diabetic animal models is required. Finally, the peptides described in this study can be used as a primary backbone to develop more potent and rationale CM-derived peptides for their beneficial and safe clinical application in diabetes.

## Supporting information

S1 File Minimal data set for Fig 1,2 and 5.
(PDF)

S2 File Original Blots for Fig 3. (PDF)

S3 File Original Blots for Fig 4. (PDF)
