# Supplementary material for: Functional profiling of synthetic camel milk-derived peptides with implication in glucose transport and diabetes
Source: PLoS One. 2025 Mar 28;20(3):e0320812. doi: 10.1371/journal.pone.0320812 (PMC11952234; doi:10.1371/journal.pone.0320812)
Supplement: S1 File — Fig 1,2 and 5. (PDF) [file pone.0320812.s001.pdf]

Log M	Insulin		
	Average	SD	SEM
-8	1.1	5.7	2.3
-7.3	11.2	10.3	4.2
-7	22.3	4.3	1.8
-6.3	51.7	4.3	1.7
-6	64.4	9.6	3.9
-5.3	80.2	7.3	3.0
-5	95.2	13.4	5.5
-4.3	95.5	8.8	3.9
-4	101.3	0.5	0.3

mg/ml	P1		
	Average	SD	SEM
0	1.6	2.7	1.6
0.001	10.1	2.8	2.0
0.005	16.5	10.1	7.2
0.01	37.6	13.2	6.6
0.05	43.0	9.7	4.4
0.1	58.5	20.3	9.1
0.5	56.7	26.3	11.7
1	55.3	16.0	8.0

mg/ml	P2		
	Average	SD	SEM
0	0.0	0.0	0.0
0.001	6.9	7.0	4.9
0.005	19.9	6.7	4.8
0.01	32.1	15.3	7.6
0.05	47.3	14.0	6.3
0.1	59.2	13.4	6.0
0.5	68.7	20.2	9.0
1	58.9	12.8	6.4

mg/ml	P3		
	Average	SD	SEM
0	0.0	0.0	0.0
0.001	6.0	8.4	6.0
0.005	11.0	0.0	0.0
0.01	19.1	1.8	1.0
0.05	48.8	18.2	8.1
0.1	47.7	31.5	14.1
0.5	46.3	15.9	7.1
1	41.4	15.9	7.9

mg/ml	P4		
	Average	SD	SEM
0	0.0	0.0	0.0
0.001	10.7	1.4	1.0
0.005	9.6	7.3	4.2
0.01	15.8	2.8	1.6
0.05	14.2	5.2	2.6
0.1	10.1	3.3	1.7
0.5	15.2	4.8	2.4
1	10.9	6.4	4.5

mg/ml	P5		
	Average	SD	SEM
0	0.0	0.0	0.0
0.001	7.3	0.0	0.0
0.005	10.1	11.9	6.9
0.01	16.7	13.6	7.9
0.05	17.9	0.7	0.5
0.1	10.8	13.5	6.8
0.5	14.9	12.6	6.3
1	20.9	6.1	4.3

mg/ml	P6		
	Average	SD	SEM
0	0.0	0.0	0.0
0.001	18.3	4.2	2.4
0.005	25.4	5.7	2.8
0.01	35.0	9.3	4.7
0.05	44.5	16.0	7.2
0.1	45.6	11.3	5.0
0.5	47.9	17.6	7.9
1	40.4	11.5	5.7

mg/ml	P7		
	Average	SD	SEM
0	0.0	0.0	0.0
0.001	7.4	3.8	2.2
0.005	26.1	18.9	9.4
0.01	31.2	19.1	9.6
0.05	44.3	24.2	10.8
0.1	40.4	16.5	7.4
0.5	50.8	15.6	7.0
1	41.1	8.4	4.2

mg/ml	P8		
	Average	SD	SEM
0	0.0	0.0	0.0
0.001	10.7	7.7	4.4
0.005	19.9	13.0	6.5
0.01	31.8	18.5	9.3
0.05	34.4	14.1	7.1
0.1	39.9	15.9	8.0
0.5	39.0	11.1	5.6
1	31.6	12.6	7.3

mg/ml	P9		
	Average	SD	SEM
0	0.0	0.0	0.0
0.001	3.3	1.8	1.1
0.005	14.7	8.1	4.1
0.01	24.7	12.9	6.4
0.05	29.3	10.8	5.4
0.1	40.2	9.0	4.5
0.5	39.3	5.0	2.5
1	31.7	15.2	7.6

Minimal Data Set for Figure 1

Treatments	Experiment 1	Experiment 2	Experiment 3	Experiment 4	Experiment 5	Experiment 6	Experiment 7	Average	SD	SEM
Basal	1.0	1.3	2.0	0.0	0.0	0.0	10.9	2.5	4.2	1.7
Vehicle	100.0	100.0	100.0	100.0	100.0	100.0	100.0	100.0	0.0	0.0
P1	73.6	47.0	66.9	125.9	98.2	190.8	122.6	103.6	48.2	18.2
P2	76.0	33.7	14.3	58.1	54.3	70.5	56.8	52.0	21.3	8.1
P3	81.5	130.9	81.8	135.0	93.0	152.3	100.8	110.8	28.4	10.7
P4	76.2	79.6	121.9	113.1	93.8	168.9	141.2	113.5	33.8	12.8
P5	25.2	59.8	53.9	134.6	95.0	147.9	130.9	92.5	47.3	17.9
P6	115.3	195.4	142.3	129.2	ND	119.3	ND	140.3	32.5	14.5
P7	ND	149.4	179.1	160.6	ND	120.3	124.3	146.7	24.7	11.1
P8	ND	ND	166.4	149.5	127.2	152.7	ND	149.0	16.3	8.1
P9	96.6	93.8	107.9	136.4	113.3	127.9	83.8	108.5	18.9	7.2

Minimal Data Set for Figure 2

Treatments	Experiment 1	Experiment 2	Experiment 3	Experiment 4	Experiment 5	Average	SD	SEM
Basal	35610	6110	88560	58390	71890	52112	32201	14401
Insulin	1246890	2847890	2370940	3140390	3006390	2522500	770060	344381
P1	1546390	3381390	790940	1217890	1409890	1669300	998617	446595
P2	1139390	2345390	1366440	1050890	1184390	1417300	531431	237663
P3	1468390	2904390	1953940	1282390	1331890	1788200	678176	303290
P4	1220390	3376390	2757940	1332890	1202890	1978100	1019159	455782
P5	2502890	4484390	1467440	1151890	1093890	2140100	1427489	638392
P6	2125890	4465390	4125940	3223390	3186890	3425500	916346	409802
P7	3674390	4084890	2424940	1551890	1291890	2605600	1244928	556749
P8	2810390	5105890	4625940	3256890	3143890	3788600	1011392	452308
P9	2272890	4201890	2672940	1456390	1205390	2361900	1188244	531399

Minimal Data Set for Figure 5
