# Supplementary material for: Functional profiling of synthetic camel milk-derived peptides with implication in glucose transport and diabetes
Source: PLoS One. 2025 Mar 28;20(3):e0320812. doi: 10.1371/journal.pone.0320812 (PMC11952234; doi:10.1371/journal.pone.0320812)
Supplement: S2 File — (PDF) [file pone.0320812.s002.pdf]

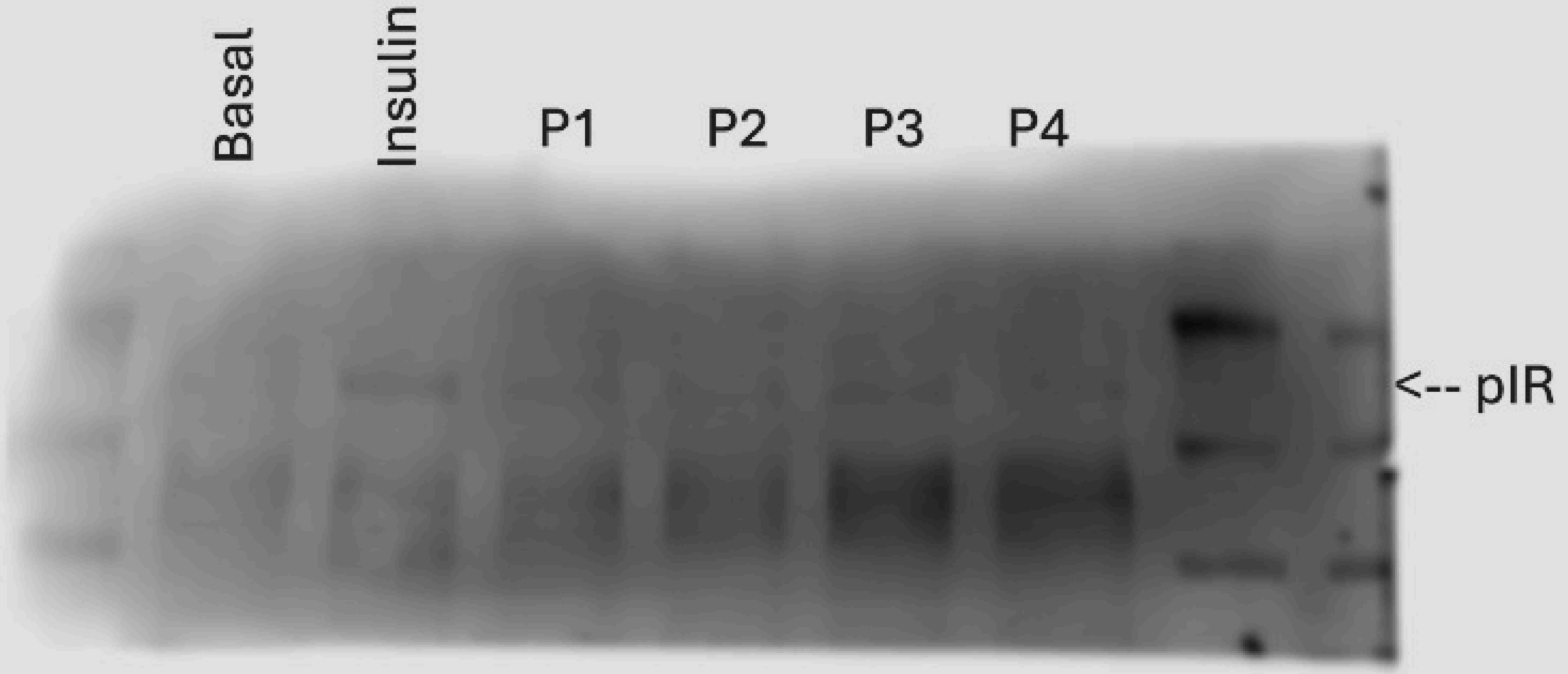

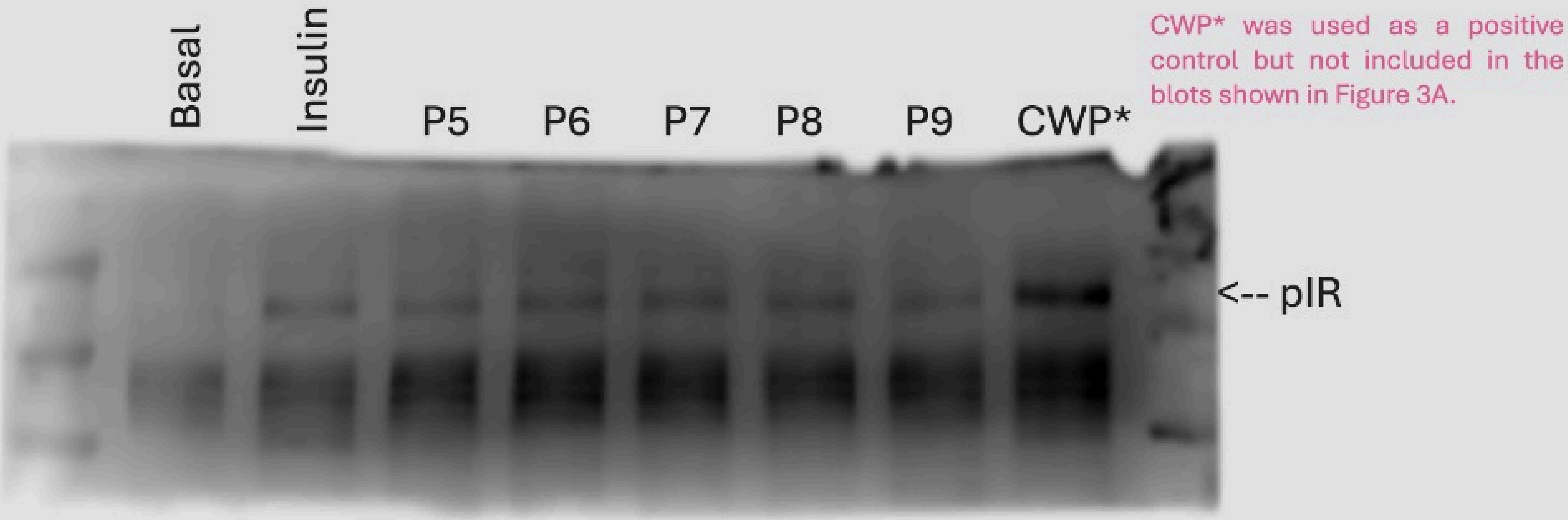

Original blots used in Figure 3A for pIR (upper blots)

Original blots used in Figure 3A for total IR (lower blots)

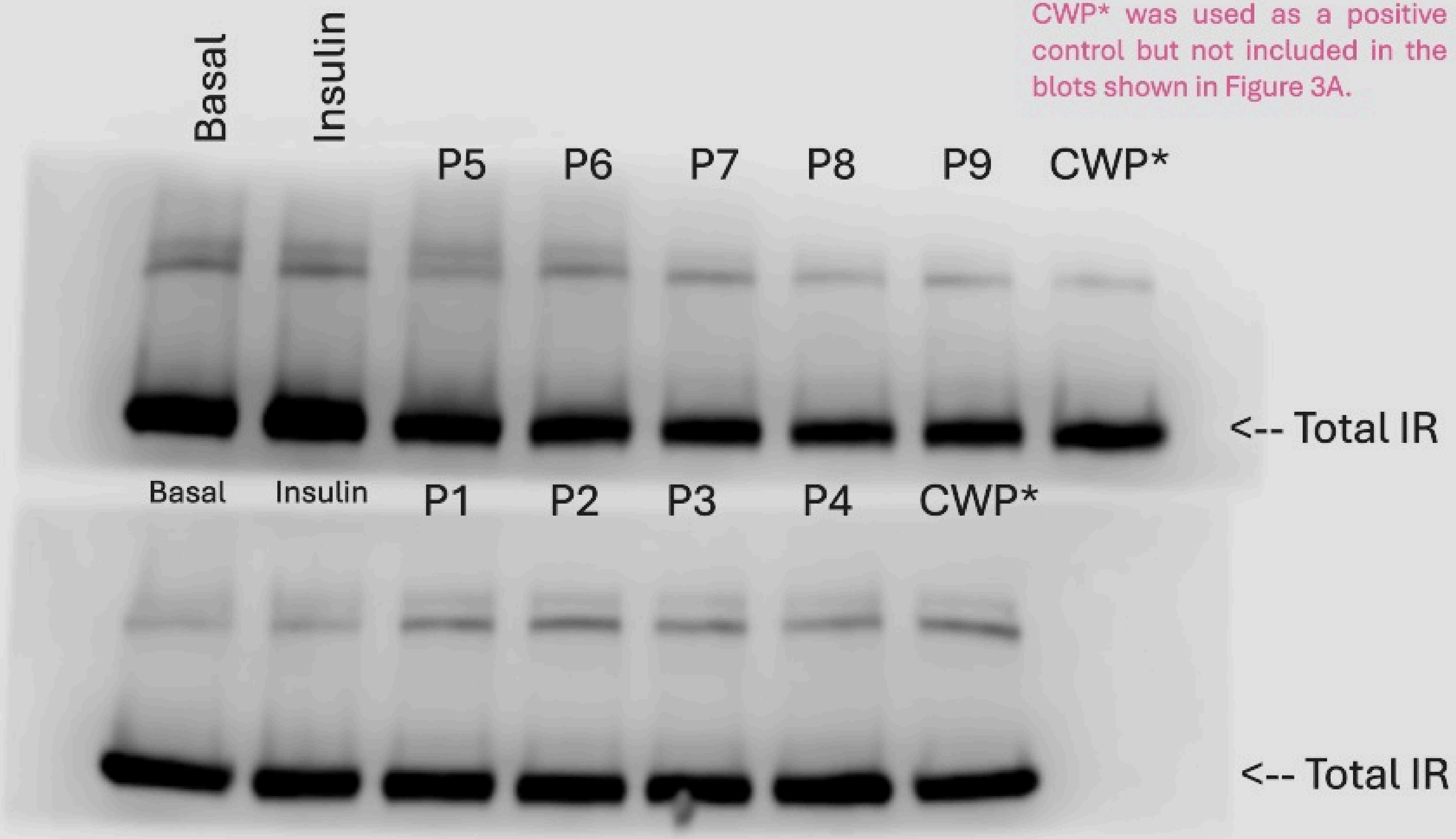

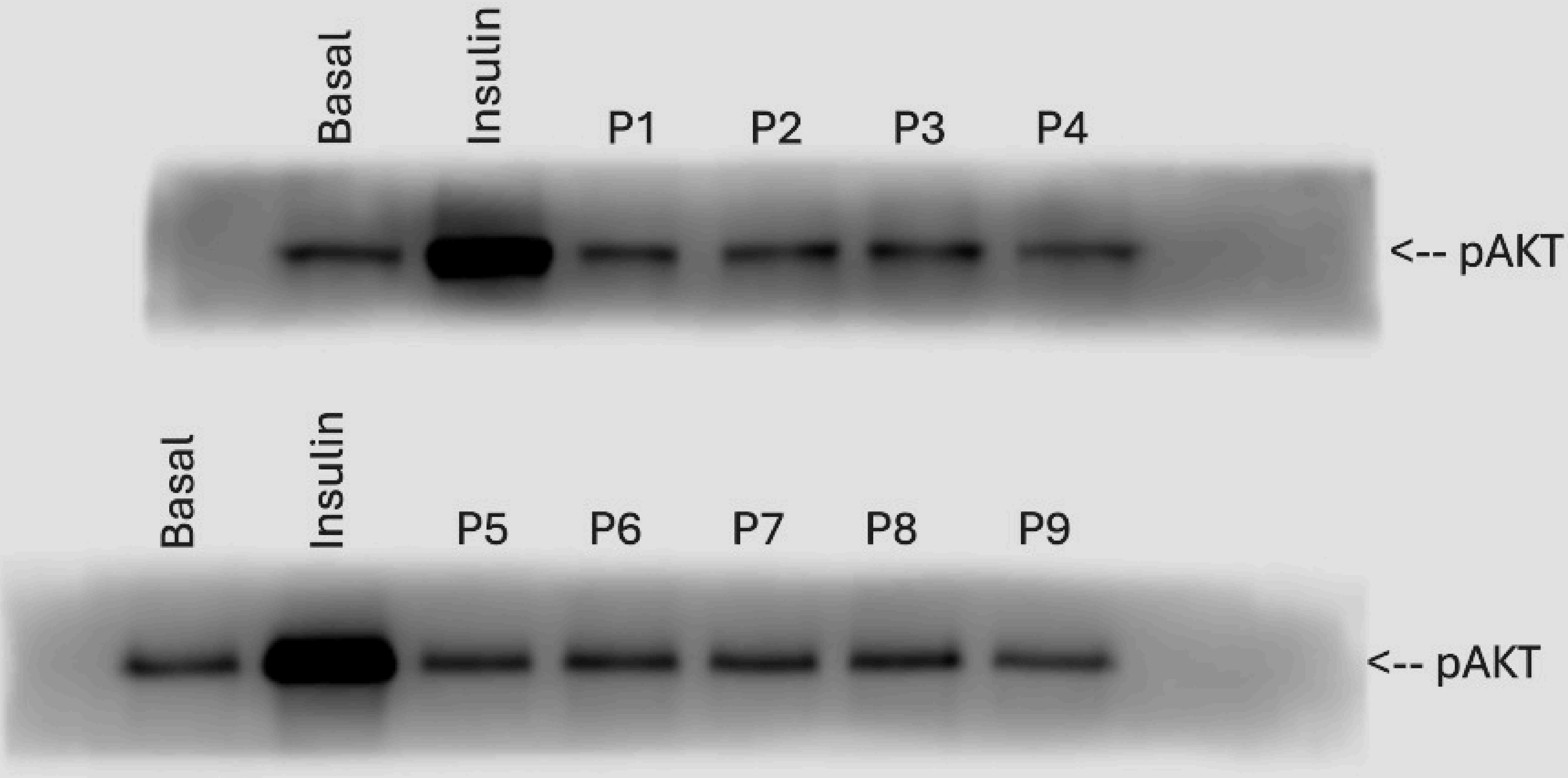

Original blots used in Figure 3B for pAKT (upper blots)

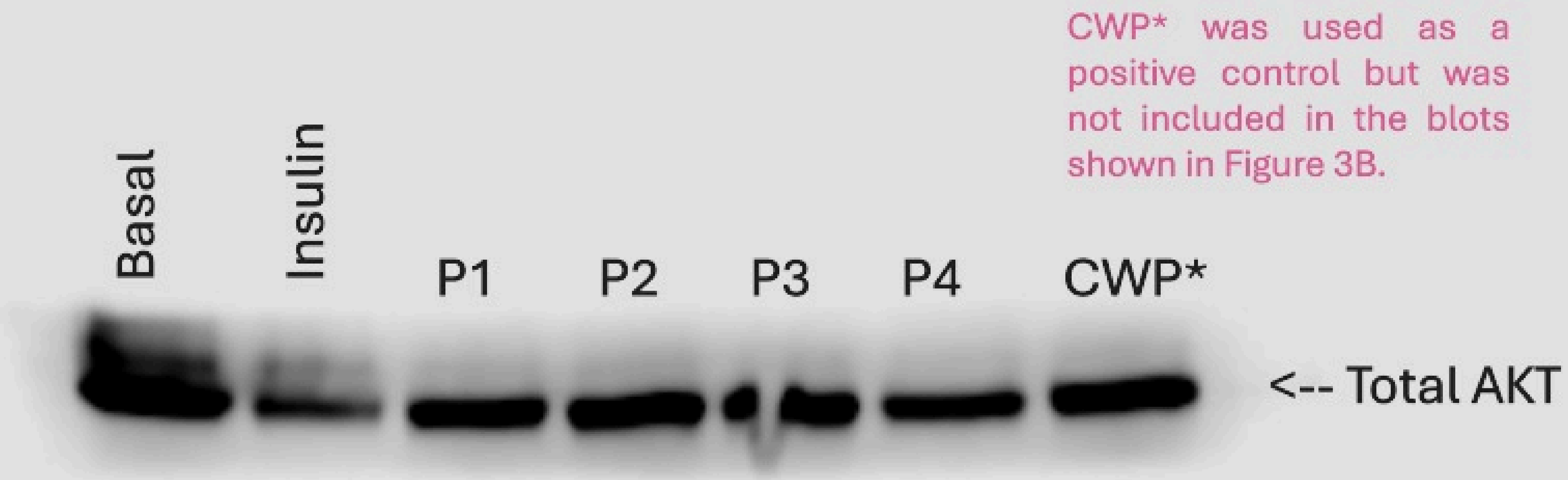

Original blots used in Figure 3B for total AKT (lower blots & left panel)

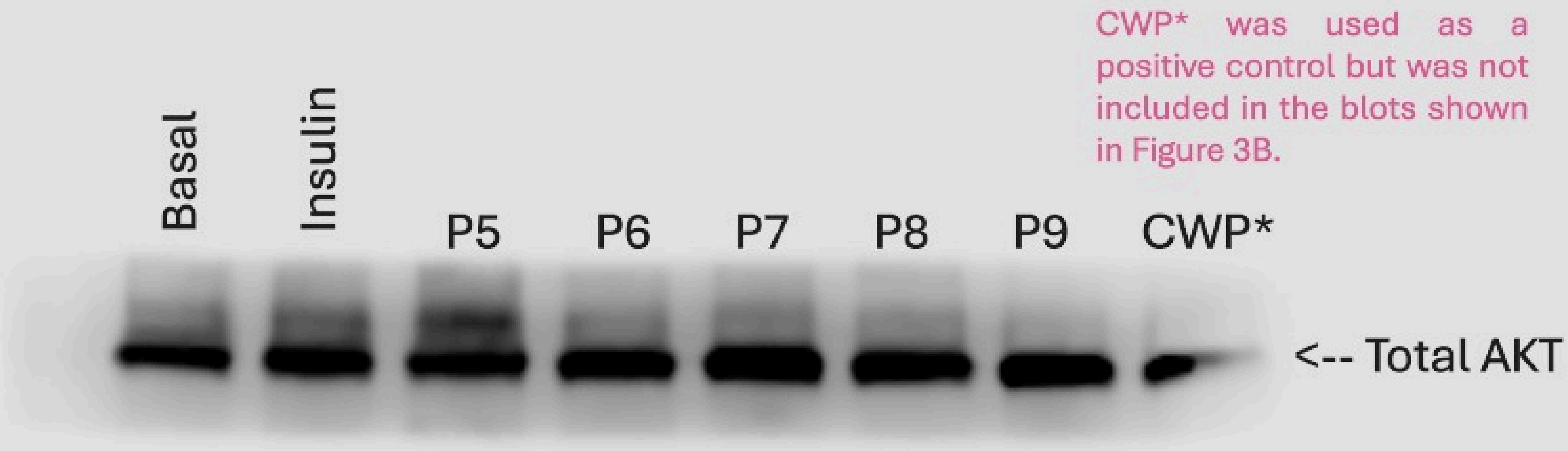

Original blots used in Figure 3B for total AKT (lower blots & right panel)
